# Vasa vasorum enhancement on optical coherence tomography in Kawasaki disease

**DOI:** 10.1038/s41390-024-03431-w

**Published:** 2024-07-22

**Authors:** Nobuyuki Kakimoto, Hiroyuki Suzuki, Akira Taruya, Takashi Takeuchi, Tomohiro Suenaga, Tomoya Tsuchihashi, Takayuki Suzuki, Shoichi Shibuta, Yasushi Ino, Atsushi Tanaka, Daisuke Tokuhara

**Affiliations:** 1 Department of Pediatrics, Wakayama Medical University, Wakayama, Japan; 2 Department of Pediatrics, Wakayama Tsukushi Medical and Welfare Center, Iwade, Japan; 3 Department of Cardiovascular Medicine, Wakayama Medical University, Wakayama, Japan; 4 Department of Pediatrics, Kainan Iryou Center, Kainan, Japan; 5 Department of Pediatrics, Kinan Hospital, Tanabe, Japan; 6 Department of Cardiovascular Medicine, Kinan Hospital, Tanabe, Japan

## Abstract

**Background:**

Patients with Kawasaki disease (KD) prone to develop coronary artery aneurysm (CAA) with unknown etiology. We aimed to disclose the relationship between vasa vasorum (VV) and intimal thickening using optical coherence tomography (OCT) in KD.

**Methods:**

Forty-three coronary artery branches of 21 patients with KD were examined by OCT. The coronary arteries were classified into three groups: the CAA group (*n* = 9) in which CAAs remained since the acute phase, the regressed group (*n* = 16) in which CAAs were regressed, and the no CAA group (*n* = 18). The number and distribution of VV, and intimal thickening in coronary arteries were evaluated on OCT.

**Results:**

Intimal thickening was significantly more severe in the CAA and regressed groups than in the no CAA group (median: 481, 474, and 218 μm, *p* = 0.001 and *p* < 0.001, respectively). The number of VV in the regressed group was significantly higher than that in the CAA and no CAA groups. The numbers of adventitial VV and internal VV were positively correlated with the intimal thickness (*R* = 0.64, *p* < 0.001; *R* = 0.62, *p* < 0.001, respectively). In the no CAA group, no internal VV were observed.

**Conclusions:**

VV enhances according to intimal thickening, suggesting that VV may have some link to the healing process, such as CAA regression and intimal thickening.

**Impact:**

- Kawasaki disease (KD) is a vasculitis syndrome developing coronary artery aneurysm, however its etiology still remains unclear. Coronary artery imaging using optical coherence tomography (OCT) can reveal coronary arterial wall pathology, however OCT studies are limited in patients with KD.
- Using OCT, we disclosed the closed relationship between vasa vasorum enhancement and regressed coronary arterial lesions.
- Vasa vasorum enhancement is involved in the pathomechanism of the convalescent phase of KD.

## Introduction

Kawasaki disease (KD) is a vasculitis syndrome of unknown etiology that mainly appears in infants. Coronary artery aneurysms (CAAs) develop in 2.6% of these patients and are recognized as a serious complication of KD.^1,2^ Optical coherence tomography (OCT) is a high-resolution (10–20 μm) method for coronary artery imaging using near-infra-red light.^3^ Recent OCT studies of KD reported that intimal thickening and an increased number of vasa vasorum (VV) were found almost exclusively in segments with a history of CAAs.^4^ However, the role of the VV in patients with CAA and KD is still unclear. Therefore, this study aimed to investigate the relationships between the VV, intimal thickening, and CAA regression using OCT in patients with the convalescent phase of KD.

## Methods

### Patients

We included 21 patients with the convalescent phase of KD who underwent follow-up coronary angiography and OCT between January 2012 and December 2016 at Wakayama Medical University Hospital. The diagnosis of KD was based on the established diagnostic criteria described in the guideline.^5^ Briefly, the patients had an aneurysm (inner diameter of the coronary artery was ≥4 mm or the Z score.^6^ was ≥2.5) in a coronary arterial branch on echocardiography at 1 month after the onset of KD. Additionally, the patients were diagnosed with a CAA using cardiac catheterization within the acute phase (median of 2 months) after onset. To follow the disease course based on the guidelines for observing KD,^7,8^ coronary angiography (CAG) was repeatedly performed. In a follow-up CAG, the OCT imaging was limited to patients weighing ≥30 kg for the reason of safety.

### CAG

To perform CAG, a 6 Fr sheath was used in the right radial artery, and 5 Fr catheters for CAG were advanced to the bilateral coronary ostia. Bilateral selective CAG was performed.

### OCT imaging

To perform OCT imaging, a 6 Fr guiding catheter was advanced to the ostium of each coronary artery. A guidewire was then advanced, and a C7 Dragonfly Intravascular Imaging Catheter (0.036-inch outer diameter; St. Jude Medical, St. Paul, MN) was inserted into the target coronary artery and pulled back. OCT images were recorded on a C7-XR OCT Intravascular Imaging System (St. Jude Medical).

### Classification of the coronary artery

Coronary arteries were classified into three groups as follows. In the CAA group, the coronary aneurysm (the inner diameter of the coronary artery was ≥4 mm or the Z score was ≥2.5) had formed in the acute phase and remained on the latest CAG in the convalescent phase. In the regressed group, a coronary aneurysm had formed in the acute phase, but was regressed in the convalescent phase and showed no abnormal findings on the latest CAG. In the no CAA group, no CAAs were documented.

### Measurement of intimal thickness of the coronary arteries

In OCT, images of three layers with high, low, and high signals were acquired in a concentric pattern outward from the vascular lumen. The high- and low-signal regions on the near side of the vascular lumen showed the intima and media, respectively, and the outside high-signal region was the adventitia. In each image, the intimal thickness was measured in four directions (top, bottom, left, and right) using image analysis software (ImageJ v.1.50i, U.S. National Institutes of Health, Bethesda, MD) and the average value was calculated.

### Definition and evaluation of VV

Signal-poor tubuloluminal structures in the adventitia or intima with continuity in at least two cross sections in the long axial direction were defined as VV as described by a previous study by Taruya et al.^9^ The number of VV was automatically counted by ImageJ v.1.50i. These measurements are based on the method used by Taruya et al.^9^ and Nishimiya et al.^10^ to assess VV using ImageJ. Regarding the position of VV, the longitudinal distribution in the adventitia and intima was defined as the adventitial VV and internal VV, respectively.

### Three-dimensional rendering of images

To visualize the VV, three-dimensional (3D)-rendered images were reconstructed with OsiriX (Pixmeo SARL, Bernex, Switzerland), as described by Taruya et al.^9^.

### Statistical analysis

Calculations were performed using JMP Pro v.14 (SAS Institute Japan, Tokyo, Japan). Categorical variables were compared by Fisher’s exact test, and continuous variables were compared by the Steel–Dwass test. The number of VV and intimal thicknesses were evaluated by single regression analysis. A *p* value < 0.05 was regarded as significant in all analyses.

## Results

### Characteristics of patients and coronary arteries

**Fig. 1:**
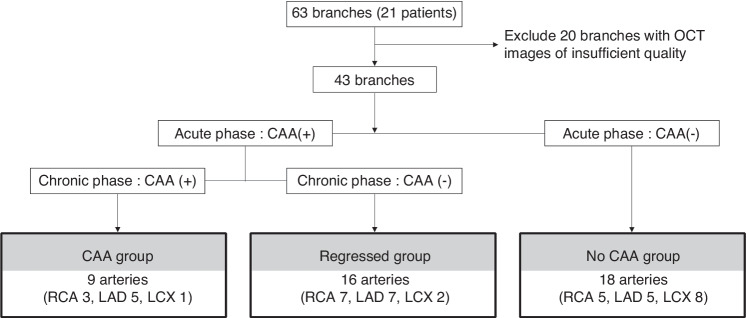
Flowchart of grouping of the coronary arteries. CAA coronary artery aneurysm, RCA right coronary artery, LAD left anterior descending artery, LCX left circumflex artery.

Twenty-one patients (boys: 16, girls: 5) were included in the current study. The median age at onset of KD was 1 y and 1 mo (0 y and 1 mo to 10 y and 11 mo), and the median age at the OCT examination was 17 y and 11 mo (13 y and 3 mo to 18 y and 6 mo). The median time from onset to the OCT study was 14 y and 5 mo (9 y and 1 mo to 18 y and 3 mo). The number of coronary lesions at the time of the first CAG was as follows: 5 with 3 branches, 7 with 2 branches, 7 with 1 branch, and 2 with 0 branches. In these two cases, all of coronary aneurysms or dilated lesions in coronary branches had regressed by the first CAG (Table 1). Sixty-three coronary arteries from the 21 patients were analyzed. Because of poor OCT images, 20 branches were excluded from the analysis. Finally, we analyzed 43 arteries (9 in the CAA group, 16 in the regressed group, and 18 in the no CAA group) (Fig. 1 and Supplementary Table 1).

**Table 1:** Clinical characteristics of the patients.

Item		Value
Number of patients		21
Male:Female		16:5
Age at onset		1 y 1 m (0 y 1 m–10 y 11 m)
The number of lesion branches	0	2^a^
	1	7
	2	7
	3	5
Age at OCT		17 y 11 m (13 y 3 m–18 y 6 m)
Interval between onset and OCT		14 y 5 m (9 y 1 m–18 y 3 m)

Values are shown as a number or median (interquartile range).

*OCT* optical coherence tomography.

^a^In these two cases, all of coronary aneurysms or dilated lesions in coronary branches had regressed by the first CAG.

The coronary artery diameter and Z score are shown in Table 2. The Z score of the coronary artery at time of the first CAG was significantly different among the groups (*p* = 0.001). Post-hoc analysis showed that the Z score was significantly higher in the CAA group and the regressed group than in the no CAA group (both *p* < 0.001). The Z score of the coronary artery at time of OCT was 4.4 (2.2–6.6), 0.7 (−1.1–1.9), and 0.3 (−1.7–1.9) in CAA group, regressed group, and no CAA group, respectively. The Z score of the coronary artery was significantly higher in the CAA group than in the regressed group and the no CAA group (both *p* < 0.001).

**Table 2:** Coronary artery characteristics.

Item	CAA group (9 branches)	Regressed group (16 branches)	No CAA group (18 branches)	*p* value	Steel–Dwass test *p* value	
Branches	RCA: 3, LAD: 5, LCX: 1	RCA: 7, LAD: 7, LCX: 2	RCA: 5, LAD: 5, LCX: 8	0.228^a^		
CA diameter (mm) at the first CAG	5.6 (4.3–7.0)	4.7 (3.6–5.6)	1.8 (1.6–2.0)			
CA Z score	6.2 (5.7–8.1)	6.2 (4.8–7.8)	1.2 (0.7–1.6)	<0.001^b^	CAA vs. regressed	0.979
					CAA vs. no CAA	<0.001
					Regressed vs. no CAA	<0.001
CA diameter (mm) at OCT	5.5 (4.6–6.4)	3.2 (2.9–3.4)	3.1 (2.6–3.3)			
CA Z score	4.4 (2.4–6.1)	0.7 (−0.1–1.4)	0.3 (−0.3–0.6)	<0.001^b^	CAA vs. regressed	<0.001
					CAA vs. no CAA	<0.001
					Regressed vs. no CAA	0.244

First CAG: CAG performed 1–3 months after the onset of KD.

Values are shown as the number or median (interquartile range).

The CA diameter and Z score were measured by CAG.

*CA* coronary artery, *CAA* coronary artery aneurysm, *CAG* coronary angiography, *RCA* right coronary artery, *LAD* left anterior descending artery, *LCX* left circumflex artery, *OCT* optical coherence tomography.

^a^Fisher’s exact test.

^b^Kruskal–Wallis test.

### Evaluation of intimal thickening and VV

A comparison of intimal thickening and the number of VV in the three groups is shown in Table 3. The median intimal thickness in the CAA and regressed groups was significantly greater than that in the no CAA group (*p* = 0.001 and *p* < 0.001, respectively). The number of adventitial VV in the regressed group was significantly higher than that in the CAA and no CAA groups (*p* = 0.0015 and *p* < 0.0001, respectively). There were no VV on the internal side in the CAA group. The number of internal VV was significantly higher in the CAA and regressed groups than in the no CAA group (*p* = 0.0021, *p* < 0.0001, respectively).

**Table 3:** Optical coherence tomographic findings of the intimal thickness and vasa vasorum.

Item	CAA group (9 branches)	Regressed group (16 branches)	No CAA group (18 branches)	Kruskal–Wallis test p value	Steel–Dwass test p value	
Maximum intimal thickness (µm)	524 (469–637)	595 (432–844)	255 (181–312)	<0.0001*	CAA vs. regressed	0.4769
					CAA vs. no CAA	0.0053*
					Regressed vs. no CAA	<0.0001*
Mean intimal thickness (µm)	481 (382–541)	474 (441–603)	218 (150–283)	<0.0001*	CAA vs. regressed	0.7918
					CAA vs. no CAA	0.0012*
					Regressed vs. no CAA	<0.0001*
Total number of VV (/section)	4.0 (3.0–6.5)	11.0 (9.3–15.8)	2.0 (2.0–4.0)	<0.0001*	CAA vs. regressed	0.0013*
					CAA vs. no CAA	0.1545
					Regressed vs. no CAA	<0.0001*
Adventitial VV	3.0 (1.5–5.5)	10.0 (7.3–12.0)	2.0 (2.0–4.0)	<0.0001*	CAA vs. regressed	0.0015*
					CAA vs. no CAA	0.8149
					Regressed vs. no CAA	<0.0001*
Internal VV	1.0 (0.0–1.5)	2.0 (0.3–4.0)	0.0 (0.0–0.0)	<0.0001*	CAA vs. regressed	0.1899
					CAA vs. no CAA	0.0021*
					Regressed vs. no CAA	<0.0001*

* : *p* < 0.05. Values are shown as the median (interquartile range).

*CAA* coronary artery aneurysm, *VV* vasa vasorum, *adventitial VV* vasa vasorum run longitudinally at the adventitia, *internal VV* vasa vasorum run longitudinally at the intima.

**Fig. 2:**
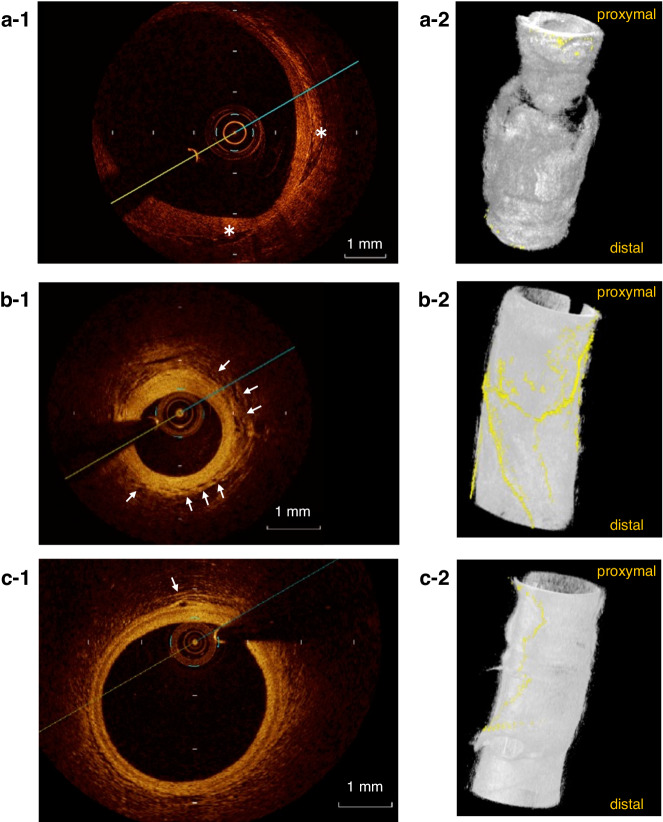
Representative OCT images. **a-1,** **a-2** Cross-sectional and 3-dimensional rendering of an OCT image of the coronary artery in the CAA group. **b-1,** **b-2** Cross-sectional and 3-dimensional rendering of an OCT image of the coronary artery in the regressed group. **c-1, c-2** Cross-sectional and 3-dimensional rendering of an OCT image of the coronary artery in the no CAA group. In the CAA group images, lamellar calcifications are observed. (asterisks in **a-1**) Vasa vasorum is scarce on the adventitial side of the residual coronary artery aneurysm. **a-2** On the other hand, in the regressed group images and the no CAA group images, vasa vasorum (arrows in **b-1**, **c-1** and highlighted in yellow in **b-2**, **c-2**) are distributed at the adventitia of the coronary arteries.

Representative 3D-rendered images of VV in each group are shown in Fig. 2. Intimal thickening and increased VV volume and branching were observed in the regressed group.

### Relationship between intimal thickness and the number of VV

**Fig. 3:**
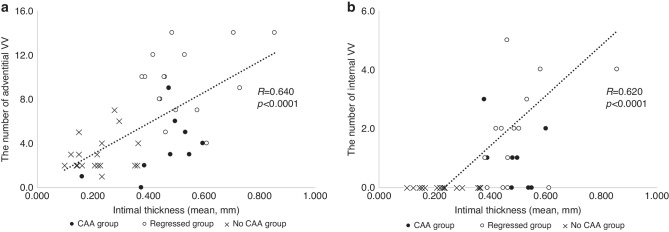
Relationship between the number of VV and intimal thickness. **a** Relationship between the number of adventitial VV and intimal thickness, **b** Relationship between the number of internal VV and intimal thickness. The numbers of the adventitial VV and internal VV were positively correlated with the intimal thickness, (*R* = 0.640, *p* < 0.0001; *R* = 0.620, *p* < 0.0001, respectively). The black dots indicate CAA Group, the white dots indicate Regressed group, and the X marks indicate No CAA group. VV vasa vasorum.

Figure 3 shows the relationship between intimal thickness and the number of VV in the adventitia and internal sides in the convalescent phase of KD. The numbers of adventitial and internal VV were positively correlated with the intimal thickness (*R* = 0.640, *p* < 0.0001; *R* = 0.620, *p* < 0.0001, respectively).

## Discussion

In the present study, we investigated the relationship between VV and coronary artery intimal thickening using OCT in the convalescent phase in patients with KD and CAA. Coronary artery branches were divided into three groups (CAA, regressed, and no CAA groups) according to the presence or absence of coronary aneurysm formation in the acute phase of KD and regression in the convalescent phase. The intimal thickness and the number of VV were examined. The following four findings were observed. First, the intimal thickening in the convalescent phase was significantly greater in the CAA and regressed groups than in the no CAA group. Second, the number of VV was significantly greater in the regressed group than in the CAA and no CAA groups. Third, the numbers of adventitial and internal VV were positively correlated with intimal thickening. Fourth, despite the absence of a significant association between coronary artery diameter in the acute phase and the regression of the coronary artery aneurysm, there was an association between the number of adventitial VV in the convalescent phase and the regression of the aneurysm.

Several autopsy studies have suggested that CAAs develop from inflammatory cell infiltration starting from the intimal and adventitial sides 6–7 days after the onset of KD.^11^ This is followed by edematous dissociation of the media, and infiltration of lymphocytes and macrophages after 10 days that advances to the internal elastic membrane and tunica media, leading to coronary arterial dilatation. From the acute phase to the convalescent stage, circumferential intimal thickening occurs, leading to the regression of CAA. In our study, the regressed group showed greater intimal thickening and a decreased Z score of the coronary artery, supporting the above-mentioned scenario. Furthermore, we found that VV were enhanced in the regressed group compared with the other groups. The VV are routes of nutrient and oxygen supply to cells of the vascular wall, and are involved in atheroma and inflammatory cell infiltration.^12^ Taruya et al. found a positive correlation between the VV and atheroma volume on OCT in adults with atherosclerotic lesions.^9^ In their study, 3D-rendered images showed VV advancing to atheroma from the adventitial and intimal sides. In particular, there was an increase in internal running of VV on the intimal side and a coral tree pattern of new blood vessels in plaques as the lesion morphologically progressed from fibrous plaques to fibroatheroma and then plaque rupture. In contrast to atherosclerotic lesions, VV in regressed CAA of KD maintains its role in supplying nutrients and oxygen to the thickened intima as a part of the regression process of CAAs. In our study, the CAA group showed a similar intimal thickness, but a smaller number of VV, than in the regressed group, which suggested that less VV formation diminished the healing process of CAAs. The location of VV might be important in the pathogenesis of CAA in KD. Hamaoka-Okamoto et al.^13^ reported that VV grew from the adventitial side before intimal thickening, which suggested that adventitial VV promoted vasculitis in KD. We also found that the adventitial VV were more enhanced in the regressed group than in the other groups. Our findings suggested that the original vasculitis still remained in the regressed group, and that vasculitis hampered the healing process of CAAs. On the other hand, as shown in Fig. 3b, the correlation is moderate, but the number of internal VVs is small in both the CAA, the regressed, and the no CAA groups, and the association is not clear. Further prospective studies are required to clarify the role of VV in the healing process of CAAs.

There are several limitations to this study. First, there were no normal controls. However, performing CAG and OCT in healthy subjects and patients is ethically impossible. Second, the duration of observation from the onset of KD to OCT varied. Patients with a relatively short observation period (5 years) were included, and this caused difficulty in observing the progression of atherosclerosis and calcification and changes in local stenosis. Therefore, a further study with a longer interval is necessary. Third, in the present study, data on the number of VVs in the acute phase and their running morphology are not available, as OCT was not performed in the CAA and regressed groups, respectively. Fourth, acquiring good-quality OCT images in large CAAs is difficult because of the shallow penetration depth of near-infrared light. Fourth, this was an observational study and our findings did not show causality between VV and the healing process of CAAs. Further molecular biological studies are required to clarify the mechanism of healing CAAs.

Severe intimal thickening in the regressed coronary arterial wall is apparent in convalescent-phase coronary arterial lesions in KD, and VV grows in the thickened region, mostly on the adventitial side. The number of VV and intimal thickening are positively correlated, suggesting that VV may have some link to the healing process, such as CAA regression and intimal thickening.

## Supplementary information

Supplemental Table 1

## Data Availability

All data generated analyzed during this study are included in this published article. For additional information concerning these data, the corresponding author can be contacted.
