# Supplementary material for: Vasa vasorum enhancement on optical coherence tomography in Kawasaki disease
Source: Pediatr Res. 2024 Jul 22;97(3):1090–5. doi: 10.1038/s41390-024-03431-w (PMC12055565; doi:10.1038/s41390-024-03431-w)
Supplement: Supplementary file 1 — Supplemental Table 1 [file 41390_2024_3431_MOESM1_ESM.pdf]

Supplemental Table 1: Characteristics of coronary arteries and OCT findings

Serial No. (43 branches)	Patient No.	Sex	Age at onset	The number of lesion branches	Type of branch	Group	CA at the first CAG		CA at OCT		OCT findings							
							diameter (mm)	Z score	diameter (mm)	Z score	Total number of VV (/section)	Adventitial VV (/section)	Internal VV (/section)	Maximum intimal thickness (μm)	Mean intimal thickness (μm)	Calcification	Disruption of tunica media	Atheroma
1	1	M	10y11m	2	RCA	CAA	4.1	3.0	4.7	2.4	3	3	0	613	548	-	+	-
2					LAD	CAA	5.8	6.0	6.0	5.9	5	5	0	1115	535	-	+	-
3	2	F	7y0m	1	RCA	No CAA	1.9	-0.7	3.2	0.5	5	5	0	181	152	-	-	-
4					LAD	CAA	5.3	6.3	5.5	4.4	1	1	0	178	162	-	+	-
5	3	M	0y6m	2	RCA	Regressed	6.8	11.1	2.9	0.6	11	10	1	485	462	-	+	-
6					LAD	Regressed	4.5	6.4	3.1	1.6	8	8	0	519	445	-	+	-
7	4	M	5y7m	2	RCA	CAA	7.3	8.7	6.6	5.3	3	0	3	471	376	+	+	-
8					LAD	CAA	6.6	7.5	6.2	6.6	4	3	1	524	481	-	+	-
9	5	M	0y1m	2	LAD	Regressed	5.8	7.8	2.7	-0.7	17	9	8	862	732	-	+	-
10					LCX	No CAA	1.8	1.9	2.9	0.4	7	7	0	319	278	-	+	-
11	6	F	9y7m	2	RCA	Regressed	4.7	5.0	3.1	0.3	16	14	2	777	486	-	+	-
12					LAD	CAA	5.6	6.1	5.3	4.3	6	4	2	660	598	-	+	-
13	7	M	0y6m	1	RCA	No CAA	2.0	1.7	3.6	0.2	4	4	0	277	232	-	+	-
14					LAD	CAA	4.5	6.2	4.5	2.4	7	6	1	551	497	-	+	-
15					LCX	No CAA	1.5	0.7	3.0	0.3	3	3	0	244	151	-	+	-
16	8	M	0y4m	3	RCA	Regressed	5.7	9.3	2.6	-1.1	27	14	13	1019	708	-	+	-
17					LAD	Regressed	6.1	7.8	3.4	1.0	14	12	2	552	420	-	+	-
18	9	M	7y3m	2	RCA	CAA	9.0	10.1	7.0	6.3	3	2	1	467	388	+	+	-
19					LAD	Regressed	5.3	5.9	3.4	2.0	18	14	4	972	856	-	+	-
20					LCX	No CAA	2.5	1.6	3.2	1.9	2	2	0	156	146	-	-	-
21	10	M	0y2m	0	RCA	No CAA	1.8	1.7	3.2	0.5	4	4	0	426	364	-	+	-
22					LAD	No CAA	1.5	0.8	2.4	-0.8	6	6	0	322	298	-	+	-
23					LCX	No CAA	1.7	1.8	2.6	0.4	2	2	0	181	163	-	-	-

24	11	M	1y2m	0	RCA	No CAA	1.9	1.0	3.2	-0.3	1	1	0	262	234	-	+	-
25					LAD	No CAA	1.9	1.1	3.4	0.5	2	2	0	523	354	-	+	-
26					LCX	No CAA	1.6	0.7	3.5	1.3	3	3	0	306	217	-	+	-
27	12	F	1y8m	2	RCA	No CAA	1.7	0.6	2.8	0.0	2	2	0	274	227	-	-	-
28					LAD	Regressed	3.0	4.5	3.4	1.7	10	10	0	434	389	-	+	-
29					LCX	Regressed	2.2	2.7	2.6	0.5	10	8	2	559	440	-	+	-
30	13	M	1y1m	1	RCA	Regressed	5.0	8.0	3.6	0.8	11	7	4	631	579	+	+	-
31					LAD	No CAA	2.0	1.5	2.8	-0.2	2	2	0	493	361	-	+	-
32					LCX	No CAA	2.0	1.3	2.1	-0.9	3	3	0	183	122	-	-	-
33	14	M	0y3m	3	RCA	Regressed	2.9	4.6	3.0	-0.3	15	10	5	540	459	-	+	-
34					LAD	Regressed	3.8	5.7	3.3	0.8	5	5	0	543	463	-	+	-
35					LCX	CAA	3.9	5.5	3.9	2.2	9	9	0	513	475	-	+	-
36	15	M	0y3m	2	RCA	Regressed	4.4	7.6	3.2	-0.2	11	10	1	427	380	-	+	-
37					LAD	Regressed	5.3	7.3	3.3	0.4	15	12	3	643	532	-	+	-
38					LCX	No CAA	1.6	1.3	3.1	0.7	2	2	0	172	143	-	-	-
39	16	M	4y2m	1	RCA	Regressed	4.7	6.1	4.1	1.6	4	4	0	888	611	-	+	-
40					LAD	No CAA	2.4	1.6	3.5	1.2	2	2	0	248	208	-	+	-
41					LCX	No CAA	1.4	-0.6	2.3	-0.5	2	2	0	113	100	-	-	-
42	17	M	0y8m	1	LAD	No CAA	1.6	0.4	2.1	-1.7	2	2	0	244	218	-	+	-
43					LCX	Regressed	3.5	4.7	3.0	0.9	9	7	2	790	502	-	+	-

CAA group: Coronary aneurysm had formed in the acute phase and remained on the latest CAG in the convalescent phase.

Regressed group: Coronary aneurysm had formed in the acute phase, but was regressed in the convalescent phase.

No CAA group: No coronary aneurysms were documented in the acute phase and the convalescent phase.

CA = coronary artery; CAA = coronary artery aneurysm; CAG = coronary angiography; RCA = right coronary artery; LAD = left anterior descending artery; LCX = left circumflex artery; OCT = optical coherence tomography.
